# Data on the modulatory effects of a single bolus dexamethasone on the surface marker expression of various leucocyte subsets

**DOI:** 10.1016/j.dib.2020.106117

**Published:** 2020-08-02

**Authors:** D.F. Draxler, C.R. Bain, R. Taylor, S. Wallace, O. Gouldthorpe, T.B. Corcoran, P.S. Myles, K. Bozaoglu, R.L. Medcalf

**Affiliations:** a Australian Centre for Blood Diseases, Monash University, Melbourne, VIC, Australia; b Department of Cardiology, University hospital of Bern, Bern, Switzerland; c Bern Center for Precision Medicine, Bern, Switzerland; d Department of Anaesthesiology and Perioperative Medicine, The Alfred Hospital and Monash University, Melbourne, VIC, Australia; e Genomics and Systems Biology Laboratory, Baker IDI Heart and Diabetes Institute Victoria, Melbourne, VIC, Australia; f Department of Anaesthesia and Pain Medicine, Royal Perth Hospital, University of Western Australia, Perth, WA, Australia and Monash University, Melbourne, VIC, Australia; g Bruce Lefroy Centre for Genetic Health Research, Murdoch Children's Research Institute and Department of Paediatrics, University of Melbourne, Melbourne, VIC, Australia

**Keywords:** Dexamethasone, Glucocorticoids, Immune response, Inflammation, Flowcytometry, Leukocytes, CDC, conventional dendritic cells, MFI, mean fluorescence intensity, PBMCS, peripheral blood mononuclear cells, Treg, regulatory T cells, SSC, side scatter

## Abstract

Dexamethasone is frequently administered to surgical patients for anti-emetic prophylaxis. We have examined the immunomodulatory effects of a single bolus of dexamethasone on circulating peripheral blood mononuclear cells (PBMCs) in the same 10 healthy male volunteers, previously used in our investigation on early *in vivo* effects of a single anti-emetic dose of dexamethasone on innate immune cell gene expression and activation [1]. Blood samples were drawn at baseline, 2 h, 4 h and 24 h. Immune cell phenotypes were examined with flow cytometry. In this data article the expression strength of markers involved in immune activation and immunosuppression as well as maturation, migration, cell death and responsiveness to signalling on monocyte and cDC subsets, as well as NK cells, CD4+ and CD8+ *T* cells and regulatory T cells (Treg) are presented. These data improve our understanding of the immunomodulatory effects of the glucocorticoid dexamethasone *in-vivo*, which may be important for the optimisation of treatment regimens as well as the evaluation of new indications for glucocorticoid treatment.

**Specifications Table**

**Table:** 

**Subject**	anaesthesiology and pain medicine
**Specific subject area**	Glucocorticoids
**Type of data**	Graphs (within manuscript) Figures (within manuscript) Table (within repository file)
**How data were acquired**	Flowcytometry of peripheral blood mononuclear cells from healthy volunteers was performed to obtain data. Stained samples were acquired on a 4-laser LSR Fortessa (BD Biosciences, San Jose, CA, USA) with BD FACSDiva software (BD Biosciences, NJ, USA). The analysis of acquired samples was performed using FlowJo data analysis software (FlowJo, LLC, Ashland, OR, USA). All statistical analyses were performed within Prism 7, Graphpad software (La Jolla, CA, USA).
**Data format**	Raw (Mendeley data repository “Data on the early in-vivo effects of a single anti-emetic dose of dexamethasone on the surface marker expression of various leucocyte subsets”, Mendeley Data, V1, doi: 10.17632/hv6v26bczp.1), and Analysed (within this manuscript)
**Parameters for data** **collection**	10 healthy male volunteers aged between 20 and 35 years. Baseline blood was collected after a 22-g intravenous (i.v.) cannula was sited. Dexamethasone (8 mg) was then injected and blood collected again at 2 h, 4 h and 24 h. The baseline and 24-h bloods were collected between 06:45 h and 1100 h. PBMCs were then isolated and analysed using flowcytometry.
**Description of data** **collection**	Surface marker expression strength on PBMCs isolated from whole blood described as mean fluorescence intensity (MFI) Cell activation/maturation: CD83, CD86, HLA-DR Cell migration: CCR4, CCR7 Inhibitory signalling; PD-L1 Programmed cell death: PD-1, CD95 Responsiveness to signalling: TNFR2, CTLA4 TGF-b release: LAP Cell viability: Zombie yellow stain for intracellular amines
**Data source location**	Institution: Australian Centre for Blood Diseases City/Town/Region: Melbourne, Victoria Country: Australia
**Data accessibility**	Data incorporated within this article and the Mendeley data repository “Data on the early in-vivo effects of a single anti-emetic dose of dexamethasone on the surface marker expression of various leucocyte subsets”, Mendeley Data, V1, doi: 10.17632/hv6v26bczp.1
**Related research article**	C.R. Bain et al.; The early in-vivo effects of a single anti-emetic dose of dexamethasone on innate immune cell gene expression and activation in healthy volunteers; Anaesthesia; doi 10.1111/anae.14306

**Value of the Data**

• We have previously reported that a single dose of 8 mg dexamethasone induces rapid and temporal changes to the immune system, affecting gene expression, cytokine levels as well as leucocyte numbers and phenotypes [1]. While in this first publication important activation/maturation markers (CD83, CD86, HLA-DR) have already been shown, we are now providing additional data on key surface markers involved in immune-activating and immunosuppressive signalling on myeloid (CCR4, CCR7, PD-L1, TNFR2) and lymphoid (PD-1, CD95, CTLA4, LAP, CCR4, CCR7, TNFR2) cell populations. The extent of immune modulation by dexamethasone is an important factor, which can now be taken into consideration by clinicians in the perioperative patient management.
• While the effects of various glucocorticoids on the immune response have been extensively studied, no comparable investigation has previously been performed to characterise the immune-modulatory changes of dexamethasone over a duration of 24 h in healthy human beings. Hence, these data will be of significant interest for researchers aiming to better understand the effects of dexamethasone, not only as to how the phenotype of various leucocyte subsets is changed, but also when those changes occur *in-vivo* after dexamethasone administration.
• An improved understanding of the extent and timing of immune modulation *in-vivo* will likely result in an optimisation of existing treatment schemes, and form the basis for exploring new indications for dexamethasone in the clinic.

## 1 Data description

**Fig. 1:**
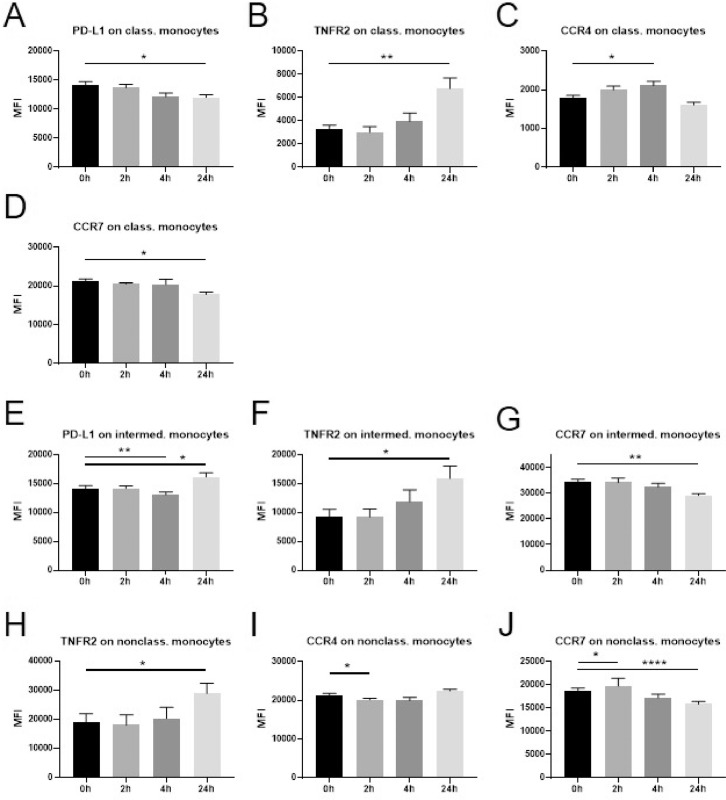
Changes in marker expression on monocytes following intravenous administration of dexamethasone (8 mg) in healthy volunteers. Flowcytometric analysis of cryopreserved PBMCs to assess surface marker expression of innate immune cells before a single injection of 8 mg dexamethasone and 2 h, 4 h, and 24 h after administration. Data are expressed as mean ± SEM; *n* = 9; RM one-way ANOVA with Dunnett's correction; * denotes *p*<0.05; ** denotes *p*<0.01; **** denotes *p*<0.0001 PBMCs: peripheral blood mononuclear cells, class. monocytes: classical monocytes, intermed. monocytes: intermediate monocytes, nonclass. monocytes: nonclassical monocytes, MFI: mean fluorescence intensity.

We examined the expression of immune-activating and immunosuppressive markers on monocyte subsets before as well as 2 h, 4 h and 24 h after dexamethasone administration (Fig. 1). On classical monocytes we found a reduction of PD-L1 at 24 h **(A)**, an increase in TNFR2 at 24 h **(B)** and of CCR4 at 4 h **(C)**, as well as a decrease in the expression of CCR7 at the 24 h time point **(D)**. In contrast, PD-L1 was downregulated at the 4 h time point yet upregulated at 24 h **(E)** on intermediate monocytes. Similar to classical monocytes, an increase in TNFR2 **(F)** and a decrease in CCR7 **(G)** was observed at 24 h for intermediate monocytes. No significant change was noted for the expression of CCR4 in this monocyte subset. Nonclassical monocytes displayed a significant increase in TNFR2 at 24 h **(H)**, consistent with classical and intermediate monocytes. At the 2 h time point CCR4 was downregulated **(I)** and CCR7 upregulated **(J)**. A decrease in CCR7 was observed at 24 h similar to classical monocytes. PD-L1 expression was not significantly influenced by dexamethasone.

**Fig. 2:**
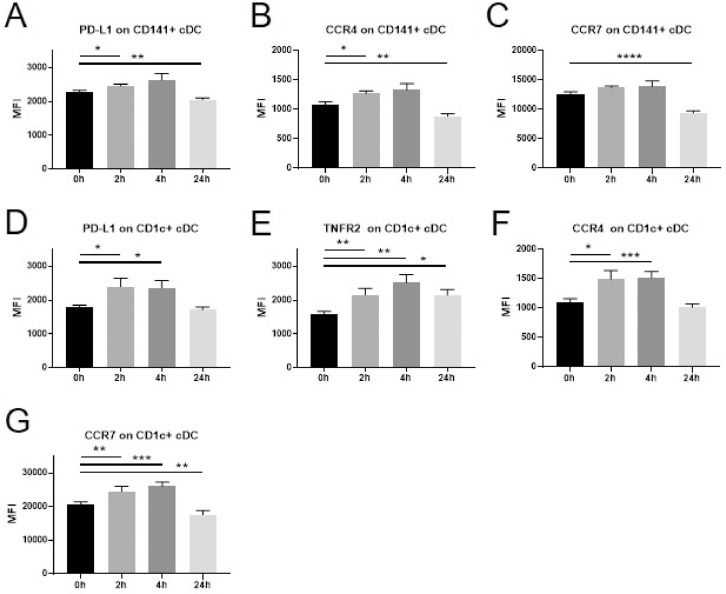
Changes in marker expression on cDC following intravenous administration of dexamethasone (8 mg) in healthy volunteers. Flowcytometric analysis of cryopreserved PBMCs to assess surface marker expression of innate immune cells before a single injection of 8 mg dexamethasone and 2 h, 4 h, and 24 h after administration. Data are expressed as mean ± SEM; *n* = 9; RM one-way ANOVA with Dunnett's correction; * denotes *p*<0.05; ** denotes *p*<0.01; *** denotes *p*<0.001 PBMCs: peripheral blood mononuclear cells, cDCs: conventional dendritic cells, MFI: mean fluorescence intensity.

We also evaluated the expression of the same markers on cDC (Fig. 2). In CD141+ cDC we detected an increase of PD-L1 **(A)** and CCR4 **(B)** at 2 h. A reduction of these markers as well as CCR7 **(C)** was observed at the 24 h time point. TNFR2 expression was not significantly altered by dexamethasone. In CD1c+ cDC dexamethasone administration induced a rapid upregulation of PD-L1 **(D)**, TNFR2 **(E)**, CCR4 **(F)** and CCR7 **(G)**. This increase was still noticeable at the 24 h time point for TNFR2, while CCR7 was downregulated at 24 h.

**Fig. 3:**
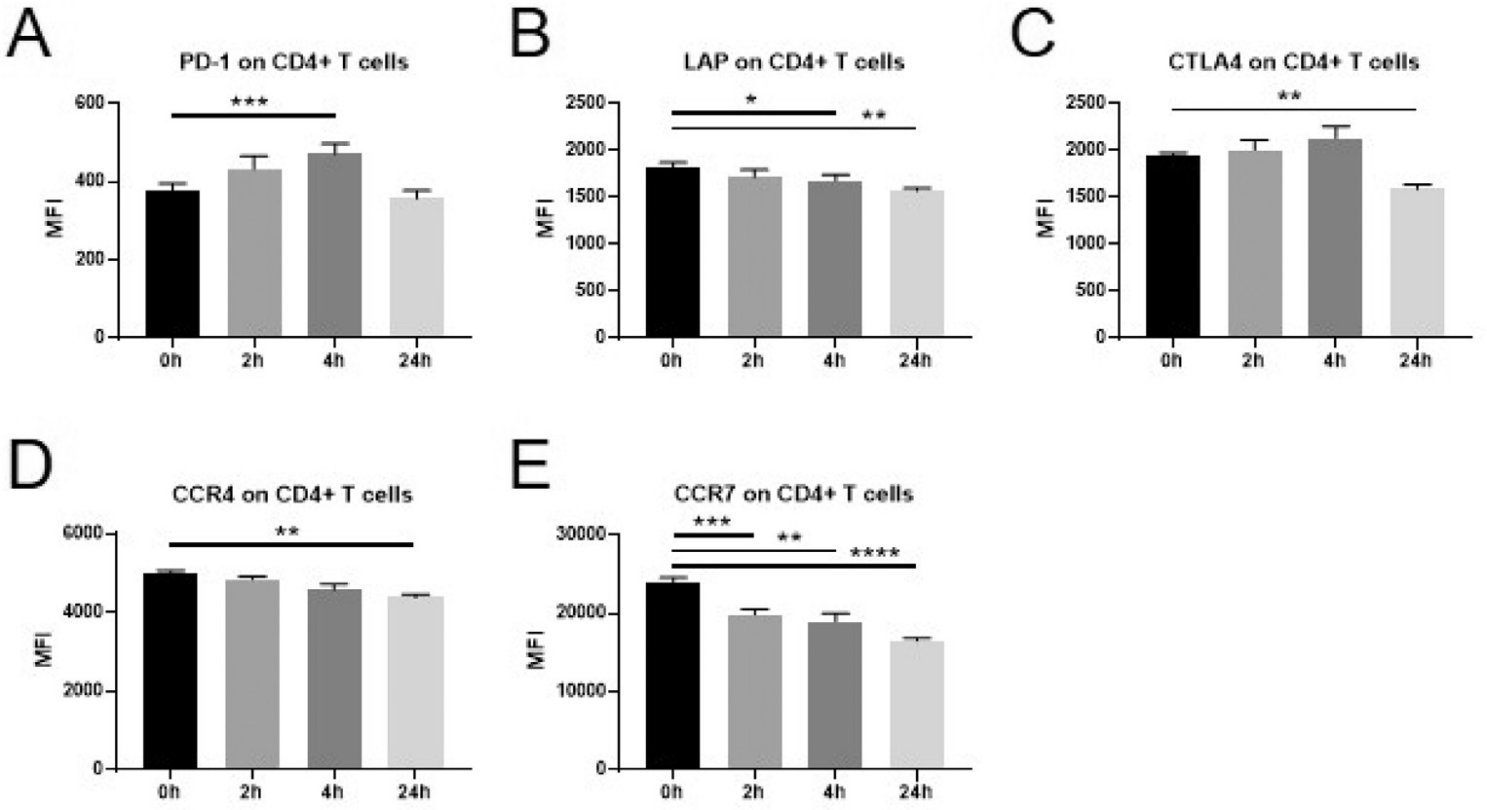
Changes in marker expression on CD4+ *T* cells following intravenous administration of dexamethasone (8 mg) in healthy volunteers. Flowcytometric analysis of cryopreserved PBMCs to assess surface marker expression of innate immune cells and T cells before a single injection of 8 mg dexamethasone and 2 h, 4 h, and 24 h after administration. Data are expressed as mean ± SEM; *n* = 9; RM one-way ANOVA with Dunnett's correction; * denotes *p*<0.05; ** denotes *p*<0.01; *** denotes *p*<0.001; **** denotes *p*<0.0001 PBMCs: peripheral blood mononuclear cells, MFI: mean fluorescence intensity.

**Fig. 4:**
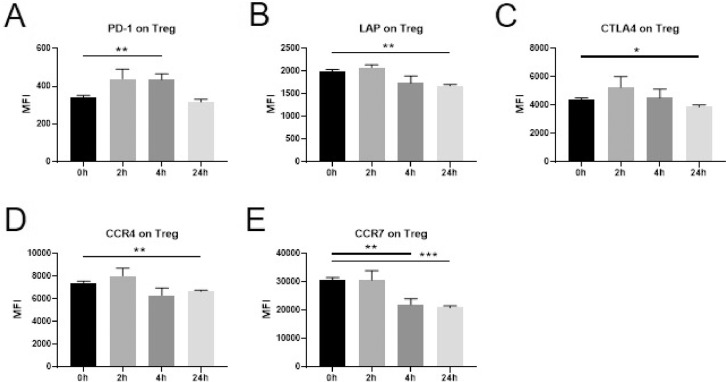
Changes in marker expression on Treg following intravenous administration of dexamethasone (8 mg) in healthy volunteers. Flowcytometric analysis of cryopreserved PBMCs to assess surface marker expression of innate immune cells and T cells before a single injection of 8 mg dexamethasone and 2 h, 4 h, and 24 h after administration. Data are expressed as mean ± SEM; *n* = 9; RM one-way ANOVA with Dunnett's correction; * denotes *p*<0.05; ** denotes *p*<0.01; *** denotes *p*<0.001 PBMCs: peripheral blood mononuclear cells, Treg: regulatory T cells, MFI: mean fluorescence intensity.

**Fig. 5:**
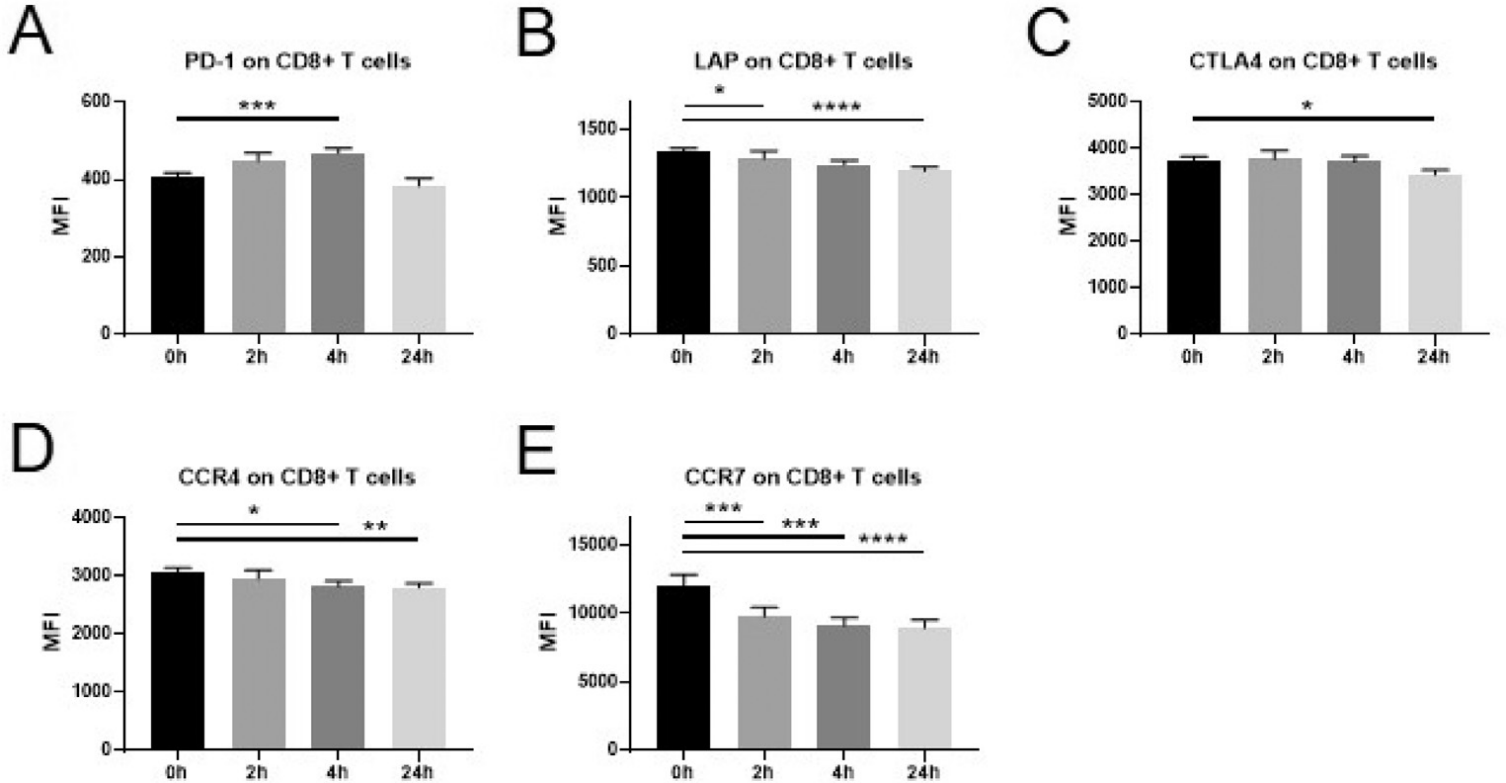
Changes in marker expression on CD8+ *T* cells following intravenous administration of dexamethasone (8 mg) in healthy volunteers. Flowcytometric analysis of cryopreserved PBMCs to assess surface marker expression of innate immune cells and T cells before a single injection of 8 mg dexamethasone and 2 h, 4 h, and 24 h after administration. Data are expressed as mean ± SEM; *n* = 9; RM one-way ANOVA with Dunnett's correction; * denotes *p*<0.05; ** denotes *p*<0.01; *** denotes *p*<0.001; **** denotes *p*<0.0001 PBMCs: peripheral blood mononuclear cells, MFI: mean fluorescence intensity.

Immune-activating and immunosuppressive surface markers were also assessed on T cell subsets. On CD4+ *T* cells (Fig. 3) a significant increase was observed for the surface expression of PD-1 at 4 h **(A)**. A gradual decrease was evident in the expression of LAP, CCR4 and CCR7 (**B,D,E)**, while a downregulation of CTLA4 was observed at the 24 h time point only **(C)**. The same changes in expression pattern were observed also when assessing the CD4+ *T* cell subset Treg (Fig. 4A-E) as well as for CD8+ *T* cells (Fig. 5A-E). No significant changes in the expression of CD95 or TNFR2 were observed in any of the T cell subsets examined.

In addition, we assessed the expression of each of these markers on NK cells, for which we did not observe significant changes in any marker at any of the time points assessed after dexamethasone administration.

Raw data for all parameters and each individual are provided in “Data on the early in-vivo effects of a single anti-emetic dose of dexamethasone on the surface marker expression of various leucocyte subsets”, Mendeley Data, V1, doi: 10.17632/hv6v26bczp.1.

## 2 Experimental design, materials, and methods

Overall, 10 healthy male volunteers between 20 and 35 years of age were enroled for the generation of this dataset. One participant was recovering from a recent viral illness and was therefore removed from the dataset (listed as CB 9 in the raw data file). Dexamethasone (8 mg) was mixed with saline (100 ml) and administered intravenously over 15 min. Blood draws were performed before administration of dexamethasone, as well as 2 h, 4 h and 24 h thereafter. The baseline and 24 h blood samples were collected between 06:45 and 11:00 h. All participants recruited completed the protocol. There were no adverse drug reactions or effects reported.

Ten millilitres of blood were collected into ethylenediaminetetraacetic acid (EDTA) tubes (BD Bioscience, Franklin Lakes, New Jersey, USA), and placed on ice immediately. For this data set 4 ml of whole blood were transferred into a 15 ml falcon tube and subjected to PBMC fractionation using the Ficoll-Paque density centrifugation protocol as per manufacturer's instructions (GE Healthcare, Chicago, USA) and allowed to cool in a commercially available freezing container (Nalgene Mr. Frosty Cryo 1 °C Freezing Containers, Thermo Fisher Scientific, Waltham, MA, USA) to achieve a cooling rate of 1 °C/min.

We have recently established two antibody cocktails for phenotyping and characterising PBMCs to assess the cellular innate immune response and T cell subsets [2]. The assessed myeloid and lymphoid cell populations as well as functional markers were selected as they represent key cells/markers involved in inflammation and immune stimulation as well as immunosuppression.

In brief, one cocktail was used to identify myeloid cell populations and it consisted of CD33-FITC, HLA-DR-APC/Cy7, CD11c-BV421, CD141-APC, CD1c-PECy7, CD14-AF700, CD16-BV510, CD123-BV650, CD83-PE/Dazzle 594, CD86-PerCP/Cy5.5, PD-L1-BV711, CCR4-BV605, CCR7-BV785, TNFR2-PE, and dead-cell-staining Zombie Yellow (all BioLegend, San Diego, CA, USA) for exclusion of dead cells. After incubation for 20 min on ice, cells were washed and resuspended in 100 ul of phosphate buffer solution (PBS)+2%FBS+1% paraformaldehyde (PFA).

The second cocktail was used for identification of lymphoid cell populations and consisted of CD3-BV510, CD4-BV650, CD8-AF700, CD56-APC/Cy7, CD25-PerCP/Cy5.5, CD95-APC, CD45R0-BV711, LAP-BV421, CTLA4-PE/Cy7, PD-1-FITC, CCR4-BV605, CCR7-BV785, TNFR2-PE, and dead-cell-staining Zombie Yellow (all BioLegend, San Diego, CA, USA). Cells were incubated for 20 min on ice, followed by a washing step before they were permeabilised in 200 ul of Fix/Perm buffer (eBioscience, San Diego, CA, USA). After incubation for 30–60 min at room temperature, cells were washed using Perm buffer (eBioscience, San Diego, USA) before 50 ul of FoxP3 PE/Dazzle594 antibody diluted in Perm buffer was added for intracellular staining of cells. Samples were incubated for 30 min at room temperature, and then washed again with Perm buffer, and finally resuspended in 100 ul of PBS. Stained samples were acquired on a 4-laser LSR Fortessa (BD Biosciences, San Jose, CA, USA) with BD FACSDiva software (BD Biosciences, NJ, USA). Single-stain controls for compensation were generated utilising UltraComp eBeads (eBioscience, San Diego, CA, USA) and the analysis of acquired samples was performed using FlowJo data analysis software (FlowJo, LLC, Ashland, OR, USA). cDC were identified as side scatter (SSC) low, CD33+ CD123- CD14- CD16- HLA-DR+ CD11c+ CD141+, and we distinguished between CD1+ and CD141+ cDC. We identified classical monocytes as SSC med CD33+ CD123- CD14+ CD16- HLA-DR+, intermediate monocytes as SSC med CD33+ CD123- CD14+ CD16+ HLA-DR+, and non-classical monocytes SSC med CD33+ CD123- CD14- CD16+ HLA-DR+.

CD4+ *T* cells were identified as SSC low CD3+ CD56- CD4+, and Treg as the CD25+ FoxP3+ subset within CD4+ *T* cells. CD8+ *T* cells were gated as SSC low CD3+ CD56- CD8+. NK cells were identified as SSC low CD3- CD56+.

All statistical analyses were performed within Prism 7, Graphpad software (La Jolla, CA, USA). The data were compared with pre-dexamethasone levels (0 h) using a repeated measure one-way ANOVA with Dunnett's multiple comparisons test. Differences were statistically significant if *p* < 0.05. The data sets were assessed for outliers with a ROUT test (*Q* = 1%). A maximum of 2 outliers was detected for some of the evaluated parameters in which case the entire participant was removed from the analysis.

We have already reported that a single dose of dexamethasone induces rapid and temporal changes to the immune system, affecting gene expression, cytokine levels as well as leucocyte numbers and phenotypes [1]. While in this first publication the expression pattern of the important activation/maturation markers CD83, CD86 and HLA-DR have already been shown, we are now providing additional data on important surface markers on myeloid and lymphoid cell populations involved in immune-activating and immunosuppressive signalling. We have performed further analysis on the acquired flowcytometry data and are now providing additional information on the early changes induced by a single dose of dexamethasone.

## Ethics statement

This dataset was generated in accordance with the Code of Ethics of the World Medical Association (Declaration of Helsinki) for experiments involving humans. It was approval by the Alfred Hospital Ethics Committee (Melbourne, Australia) and all participants provided written informed consent for inclusion.

## Declaration of Competing Interest

The authors declare that they have no known competing financial interests or personal relationships which have, or could be perceived to have, influenced the work reported in this article.

## Appendix Supplementary materials

Image, application 1
